# Tobacco or Healthy Children: The Two Cannot Co-Exist

**DOI:** 10.3389/fped.2013.00020

**Published:** 2013-08-23

**Authors:** Philip Keith Pattemore

**Affiliations:** ^1^Department of Paediatrics, University of Otago, Christchurch, New Zealand

**Keywords:** tobacco, passive smoking, children, environmental risk, ethics

## Abstract

Tobacco exposure increases mortality and morbidity of the fetus, the child, the adolescent, and their children in turn. Nearly half the children in the world are exposed. Smoking is not merely personal choice or personal responsibility; those subtle phrases undermine those who have no choice in the matter. Tobacco control must take a multi-pronged attack. Smoking cessation by adults in childbearing years must take center stage of these efforts, because it is the only way to ensure a smoke-free environment for children. Smoke-free parents provide a role model for smoke-free young people, and erode the image of smoking as a desirable adult behavior to emulate. Pediatricians and pediatric pulmonologists have a key role to play here. This goal will reduce morbidity and mortality among adults and children. Legislation regarding taxation, environments, tobacco constituents, product placement and display, packaging, and media education are all key to this core goal. Smoke-free policy must be protected from attack based on trade agreements. Research is needed into more effective ways to attract and help people give up smoking, and into educating and re-deploying tobacco industry workers in emerging and developed countries.

“A custome lothsome to the eye, hatefull to the Nose, harmefull to the braine, dangerous to the Lungs” [*sic*]

Since 1604 when James I of England wrote the above description of tobacco smoking, people have recognized the common sense link between smoking and disease, conclusively documented by Doll and Bradford Hill (1) in the British doctors’ study. In 1967 the first effects of passive smoking on children were sought (2), but it was not until 1981 that Hirayama’s study of non-smoking women showed that passive smoking was a serious risk to health (3). The massive extent and prevalence of disease attributable to tobacco smoke exposure in children has slowly emerged and continues to be documented (Table 1). Cigarette smoke contains many carcinogens and cellular poisons that are especially high in sidestream smoke (4), but nicotine itself is a neural teratogen, a genotoxin and potentially a carcinogen in its own right (5–8).

**Table 1:** **Increased fetal or child morbidity and mortality risks that have been associated with exposure to tobacco smoke (indicative, not exhaustive)**.

**PRE-/PERI-CONCEPTIONAL EFFECTS OF FATHER SMOKING**
Anorectal malformations [pOR 1.53 (40)]
Childhood cancers [including ALL and AML (41–45)]
**PRE/PERI-CONCEPTIONAL EFFECTS OF MOTHER OR BOTH PARENTS SMOKING**
Hepatoblastoma [mother smoking OR = 2.68, both parents OR = 4.74 (46, 47)]
**IN UTERO EFFECTS ON FETUS**
[aOR = 2.11 (48 ), fetal death, and stillbirth (pooled RR 1.26) (49 )]
Restricted fetal growth and low birth weight [pooled RR 1.82 (49)]
Alteration of development of fetal airways (50)
Cleft palate (51)
**IN UTERO EFFECTS AFFECTING POSTNATAL LIFE (52)**
Reduced respiratory drive and arousal responses in infant (50)
Sudden unexpected death of infancy [pooled aOR = 2.25 (53 )]
Hospitalization in infancy [aOR = 1.52 (54 )]
Invasive meningococcal disease [pOR = 2.93 (55 )]
LRI and bronchitis in young children (50)
Infant wheezing [aOR = 4.9 (56 ), pOR = 1.4 (57)]
Asthma [ ≤2 years pOR = 1.85; 5–18 years pOR = 1.23 (57)]
Asthma in adolescent girls [aOR ∼2 (58 )]
Decreased lung function in adolescent boys (59)
Reduced response to inhaled corticosteroids in children with asthma (60)
Learning difficulties, behavioral problems, and ADHD (61)
Sensorineural hearing loss [aOR = 1.83 (62 )]
Gestational diabetes in females (63)
Obesity [aOR 1.5–2.65 (64–66)]
Pyloric stenosis [aOR 2.0 (67)]
Smoking initiation [OR 2.1–2.7 (68)]
**POSTNATAL EXPOSURE EFFECTS ON YOUNG CHILDREN**
Sudden unexpected death of infancy [pooled independent aOR = 1.97 (53 )]
Respiratory tract infections including pneumonia, bronchiolitis, bronchitis, pharyngotonsillitis, sinusitis, otitis media, and the common cold [1.5- to 4-fold risks (61, 69)]
Increased severity of influenza (70)
Invasive meningococcal disease [pOR = 2.26 (55 )]
Wheezing [ ≤2 years pOR 1.7, 5–18 years pOR = 1.2–1.4 (57)] asthma [ ≤2 years pOR = 2.47 (57)); pOR = 1.32, pooled aOR 1.27 (71)
High blood pressure (72)
Learning difficulties, behavior problems, and ADHD (61)
Childhood and adult cancers (43, 47)
Increased severity of asthma (73–76)
Decreased pulmonary function (57, 59, 77–79)
Injury from house fires (24)
**EXPOSURE IN LATER CHILDHOOD AND ADOLESCENCE**
Respiratory infections, severe asthma, and decreased pulmonary function (as above)
Diastolic blood pressure [aOR = 2.25 (80 )]
Adverse changes in serum lipids (81, 82)
Developing the metabolic syndrome [aOR = 4.7 (83 )]
Smoking initiation (11, 12)

*OR, odds ratio; aOR, adjusted odds ratio; pOR, pooled odds ratio; RR, relative risk*.

The tobacco industry maintained there was doubt about the links of tobacco smoking with lung cancer and other health concerns, and denied the addictiveness of cigarettes into the 1990s. However, once the internal memos of tobacco companies were forced into the public arena by landmark court cases, they were compelled, on the surface at least, to admit some of the harm of smoking (while continuing to this day to indicate there is doubt about key findings). They framed this damage in terms of personal choice and personal responsibility. People knew the risks and they chose the pleasure of smoking in spite of this; the industry seems almost to imply it has a responsibility to continue to supply this legal consumer demand.

## Children Do Not have a Personal Choice

Addiction, passive smoking, and the risks to children totally negate the argument of personal choice and personal responsibility. A parent who is addicted to nicotine will find it extremely difficult not to expose their child to smoking, even if they smoke outside the home (9, 10). And that child has no choice about whether they are exposed. The fetus and the young child are the most vulnerable to, and the least able to avoid, the health consequences of exposure to smoking by their parents. Older children and teenagers are, in addition, vulnerable to the role modeling of smoking by their parents (11, 12), and to the attractive portrayal of cigarettes as a badge of adulthood. Every day nearly 100,000 young people worldwide start smoking (13). In turn, this group becomes the next generation of smoking parents.

Smoking exposure in the womb and in childhood increases risk factors for adult disease, aside from the risk of the child becoming a smoker themselves. These include (see Table 1) increased blood pressure and serum lipids (cardiovascular disease), decreased pulmonary function, and increased asthma severity (asthma and COPD), and genotoxic effects which may increase the risk of adult cancers.

So-called third-hand smoke has potential health effects on children. Third-hand smoke is when smoke is adsorbed onto clothing or fabric, and volatile substances then “off-gas” into the air again. This gas contains significant amounts of carcinogens and toxins (14). This means that smoking outside the house does not remove the risks to children and can only be recommended if it is a step toward quitting. Third-generation effects have been documented, in which tobacco-induced DNA alterations (methylation or mutations) occur in a smoker’s germ cells, and are passed on via the germline to their children and grandchildren (15, 16).

Pregnancy and childhood are thus periods when one person’s smoking intensely and intimately exposes other highly vulnerable people, and perpetuates a transgenerational cycle of smoking initiation. For these reasons elimination of smoking in pregnancy and in parents is an absolutely key issue for the immediate and future health of the population, and for reduction of uptake of tobacco smoking by the young.

## A Matter of Global Injustice and Environmental Concern

Whereas once cigarette smoking was the luxury of the elite, it is now heavily overrepresented in lower socioeconomic, and disadvantaged minority groups, who have the least resources to be able to cope with the health effects, or advocate on their own behalf. Tobacco marketing has targeted these groups (17–19).

The inequity of exposure and health effects extends to the international and global scene. Tobacco companies, like many other industries, have exploited the emerging and developing countries of the world in China (the world’s largest tobacco-growing nation), India, Africa, and South America. Tobacco has been offered as a quick cash crop to peasant farmers who become locked into dependence on the industry supply of fertilizers, and often enter a debt trap, because tobacco impoverishes the soil (20). In 1999 it was estimated that over 200,000 hectares of forest or woodland were cleared every year in developing countries for tobacco plantations and to fuel the curing of tobacco (21, 22). And thousands of children are employed in unsafe and toxic conditions in tobacco manufacturing plants and in the extensive home-based manufacture of bidis (cheap cigarettes) in India, China, and other countries (13, 23, 24).

For these reasons tobacco use is a global cause of injustice and environmental concern, and is not just an issue of personal health (25). The WHO Framework Convention on Tobacco Control (2003) required signatory nations to meet key core principles and objectives from February 2005 (26). Hard-hitting legislation – including increased taxation, the banning of sales to minors, the banning of smoking in workplaces, restaurants, bars, casinos, the removal of vending machines and tobacco point-of-sale displays, requiring package health warnings and media campaigns – has been effective in reducing smoking rates in many countries to levels below 20%. In 2007, Frieden and Bloomberg estimated that reduction of smoking prevalence from 25 to 20% worldwide would prevent 73 million premature deaths in adults, 50 million deaths in children, and 50 million antenatal deaths by 2030 (27).

Legislation on the table for many countries includes plain-packaging (following Australia’s lead), banning smoking in cars carrying children, in parks and beaches, and regulation of tobacco constituents^1^. There is evidence that these approaches will further reduce smoking levels. Aotearoa/New Zealand has committed to being a smoke-free country (defined as a smoking prevalence of less than 5%) by 2025.

## The Womb and the Home – Still Unprotected

And yet, as Tobacco Endgame conferences presage the demise of tobacco sales and smoking, the greatest area of harm to children remains the womb and the home, where legislation’s arm often falls short. Almost half of the world’s children who never smoked are exposed to tobacco smoke in the home (13). Many countries have legislated against physical punishment of children, and against neglect or leaving children younger than a certain age on their own at home. Can the home be protected from smoke by legislation, and how will it be regulated? We know that smoking outside the house does not protect children (9). We also know that measures directly addressing education of young people and teenagers are often not effective on their own in preventing smoking initiation (28). The only effective way to reduce smoking exposure in the home is for parents to give up smoking. Similarly the best way to change the image of tobacco from being seen by young people as a desirable badge of adult, independent status is for adult smokers in their childbearing years to give up smoking. We need continuing research efforts into the best way to counter media advertising, provide positive media messages, and make smoking cessation attractive as well as feasible to the majority of smokers.

## From Corporate Social Responsibility to Challenging Public Health Legislation

The other major challenge that is being faced comes from a new tactic of the tobacco industry. Since the early 90s the industry has often tried to portray itself as a caring, community-responsible enterprise that has no wish to advertise to children or young people and supports educational activities. For example a tobacco company produced and distributed an education package for schools in Australia and New Zealand on responsible decision-making (“I’ve got the Power”), and another is a sponsor for the Keep New Zealand Beautiful trust. Through such initiatives (part of the public relations front termed “Corporate Social Responsibility” (29, 30)) the industry has tried to maintain this image, despite continuing to market a deadly product, and to use product placement in movies attractive to young people. Part of this has been an industry tactic to emphasize that smoking is an adult behavior, with the double effect of appearing to discourage youth smoking while doing the opposite, because youth seek to emulate adult behaviors (31).

*“Acting in a manner that draws the clearest, sharpest possible line between who should and who should not have access to cigarettes will reinforce the right of adults to obtain and enjoy a legal product, and thus prevent marketing bans down the road that are driven by the youth access issue.” (Speech Presented at Philip Morris Invitational in 1995* (32)).

Now, as plain-packaging legislation has been enacted in Australia, and is planned in many other countries, including the UK and New Zealand, the tobacco industry has started to use tactics directly opposed to public health and the public good. Big Tobacco is pitting its massive financial might against governments through trade agreements, alleging threats to the industry’s profits and trading based on their intellectual property – including their logos and package design (33). This is clearly an area that bites deeply. Challenges have been brought to Australia based on the legislation being unconstitutional (this challenge has been overturned by the Australian High Court), being a technical barrier to trade (an action brought by several countries through the World Trade Organization, with legal support from tobacco companies) and being in breach of a longstanding trade agreement between Australia and Hong Kong (an industry shifted their office to Hong Kong in order to bring this dispute).

Australia has commendably not backed down on the legislation and has good reasons for defeating the challenges. But this will not be achieved without considerable legal expense (predicted to exceed AUD $10 million), and this has already resulted in a “chilling” effect – a delay in the planned introduction of plain packaging by the New Zealand government, and other governments (34). New Zealand and several other Pacific nations are at the same time negotiating a “new generation” trade agreement which is predicted to have a much greater reach into domestic policy. The Trans Pacific Partnership is backed by the US Foreign Trade Representative, and several tobacco companies have already intimated that they are prepared to use such an agreement to litigate to protect their investments (including intellectual property such as logos and packaging) if necessary (33–35). Tobacco companies and tobacco-growing states have major influence in US trade, regardless of the denouncement of tobacco by the US Surgeon General. It is essential that tobacco control strategies and the medical and legal professions work together to advocate against the loss of democratic powers and public health muscle that are potentially put on sale for the sake of trade.

## An Unethical Industry Can be Run by Decent People with a Blind Spot

The tobacco industry is unethical and immoral in that it continues to manufacture and market a lethal, addictive, and child-damaging product in full knowledge of these effects. The industry thus displays a disregard for human life, for the rights of children, for the status of the poor, and for the environment. Tobacco corporations have made duplicitous statements in public and in their public relations efforts that have been termed “Corporate Social Responsibility” as noted above. This is not to say that every worker in the industry is corrupt, but the industry and its corporate entities exhibit this unethical behavior and ethos. Many tobacco industry workers may have blind spots in regards to the medical, social, and environmental damage caused by their company. The controlling story within tobacco companies is not that of an unethical business producing a lethal and child-damaging product, but rather of a thriving business (despite attacks from fringe medical scientists) that benefits the community and country, treats its workers well and offers good job security, much like a food or pharmaceutical corporation (36). There has been little research into how tobacco industry employes may be best educated about the damage to the public. One interfaith group has bought industry shares in order to challenge and influence company policy, with some success (37).

**Figure 1:**
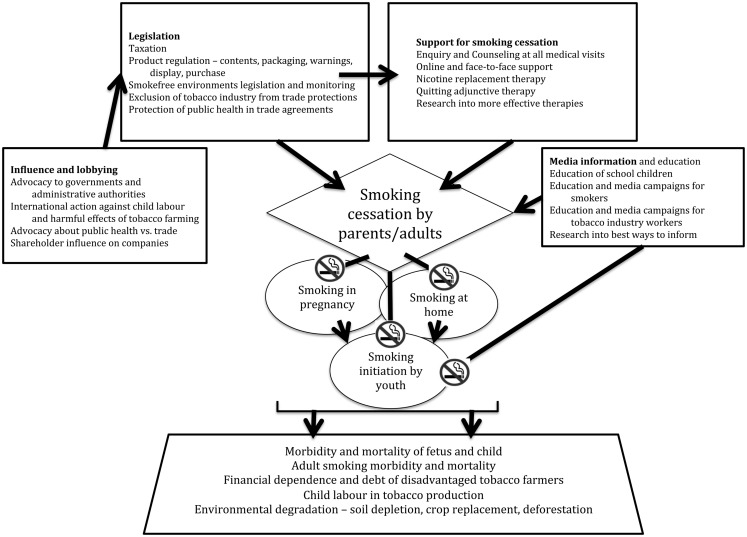
**Multi-pronged approach to reducing tobacco-related mortality, morbidity, and smoking initiation among children, focused on reducing smoking prevalence in adults**.

A multi-pronged tobacco control strategy continues to be required (Figure 1) because of the many ways in which the industrial and legal might of the tobacco industry can be brought to bear to circumvent, penetrate, or challenge smoke-free measures and legislation. As child health professionals, our goal is that every child experiences their right to grow up in a smoke-free environment.

At the same time we must never assume that the tobacco industry is going to roll over and surrender, or transmute into a charitable agency.

## The Ongoing Fight with a Formidable Opponent

The tobacco companies have more economic and legal might than many countries. In countries like America and China they have entangled themselves in every stratum of politics and economics, from farmer benefits, to states, to congress and government trade agencies (36) (although their proportional contribution to these countries’ economies may be overstated (38)). The WHO estimates that the number of smokers worldwide will increase from 1.1 to 1.6 billion by 2025 (13). So what hope do we have? Are we condemned after each public health gain to expect ever-augmented strength from the industry?

The outcome cannot be predicted with certainty, but our stance as child health professionals and respiratory specialists is clear. We must expose and oppose the injustice to the young and the marginalized that we see on a massive and largely unappreciated scale. What we do has ramifications beyond just tobacco. The alcohol industry has been following the tobacco industry’s strategies closely, emphasizing personal responsibility and targeting the “problem drinker” rather than the industry taking responsibility for its own product and marketing to youth. Other industries that are operating at the cost of health interests may follow.

What counts in our favor is that we have a transparent agenda that is in the child’s and the community’s best interests, that is devoid of vested interests, that truly captures the moral high ground, and has the support of the international community, WHO and most of our governments (albeit that governments are always having to deal a large hand to trade interests). As with those who opposed the slave trade, we cannot underestimate the strength of the financial and corporate interests opposing change, but we have a responsibility to act and refuse to stand down on behalf of those who cannot advocate for themselves. This is a war that can be won, but it requires major commitment on our part, ongoing research into key aspects of tobacco control, and ongoing dialog with our health departments and governments.

To conclude, it is naturally of direct relevance to pediatric pulmonologists that tobacco smoke from conception onward has major effects on the development, functioning, and wellness of the entire respiratory system, and through the respiratory system on sudden infant death syndrome, meningococcal disease, and many other systemic diseases. It poses a long-term risk for adult chronic cardiorespiratory disease, and for smoking initiation, with its own incumbent risks. As well as this, we should be aware that growth and manufacture of tobacco products pose global risks to the health and wellbeing of children.

For the practicing pediatrician and pediatric pulmonologist it is vital that we consistently ask parents and older children about smoking. For parents and expectant parents who smoke it is helpful to ask about their willingness to quit, to advise them strongly to quit (in a brief sentence), and to provide them with verbal and/or written information about the risks to their children and even grandchildren of exposure. Brief motivational interviewing is an accepted method of doing this (39). Children and adolescents who are smokers are more difficult to advise and counsel effectively: helpfulness and clear advice to quit are important.

Parents who are smokers and are willing to quit should be reminded of the positive immediate aspects of quitting (improved child health, improved breath, taste, fitness etc.), encouraged to set a quit date, and provided with nicotine replacement. Referral to a smoking cessation provider should be offered, and follow-up arranged. For those who relapse they should be congratulated for trying, encouraged that relapse is part of the process of quitting, and assisted to learn from their attempt.

As expert leaders in our organizations and communities we should endeavor to speak up about the impact of tobacco exposure and marketing on our children, and the impact of the industry on the environment and children in the developing world. This may involve discussing the effects of tobacco smoke with colleagues such as obstetricians, input into smoke-free initiatives, letters to the media or to MPs, and lobbying of governments for protection of children through legislation. We should also be asking our governments about the effects of their trade relationships with the tobacco industry or tobacco exporting states on public health and on global injustice to children and families.

## Conflict of Interest Statement

The authors declare that the research was conducted in the absence of any commercial or financial relationships that could be construed as a potential conflict of interest.

## Footnotes

^1^Note that harm reduction strategies that involve substituting cigarettes with smokeless forms of tobacco (electronic cigarettes etc) rather than cessation are not ideal because of the toxicity of nicotine itself to the fetus, to the child, and to the germline. See paragraph 1.
